# Tailored synthesis and optimization of nickel-molybdenum carbide-graphite nanofiber composite for enhanced ethanol electrooxidation

**DOI:** 10.1371/journal.pone.0308365

**Published:** 2024-10-03

**Authors:** Nasser A. M. Barakat, Hassan E. Gomaa, Khalil Abdelrazek Khalil, Fahad S. Al-Mubaddel, Mohamed K. Hassan, Marwa M. Abdel-Aty

**Affiliations:** 1 Chemical Engineering Department, Faculty of Engineering, Minia University, El-Minia, Egypt; 2 Department of Chemistry, College of Science and Humanities, Ad-Dawadmi, Shaqra University, Shaqra, Saudi Arabia; 3 Department of Nuclear Safety Engineering, Nuclear Installations Safety Division, Atomic Energy Authority, Amman, Egypt; 4 Department of Mechanical & Nuclear Engineering, College of Engineering, University of Sharjah, Sharjah, UAE; 5 Department of Chemical Engineering, College of Engineering, King Saud University, Diriyah Campus, Riyadh, Saudi Arabia; 6 Production Engineering & Design Department, Faculty of Engineering, Minia University, El-Minia, Egypt; 7 Mechanical Engineering Department, College of Engineering and Islamic Architecture, Umm Al-Qura University, Makkah, Saudi Arabia; Washington State University, UNITED STATES OF AMERICA

## Abstract

A novel nickel-molybdenum carbide-graphite nanofiber composite is introduced as an electrocatalyst for ethanol electrooxidation. The proposed nanofibers have been prepared by calcinating electrospun nanofibers composed of nickel acetate tetrahydrate, molybdenum chloride, and polyvinyl alcohol. The calcination process was conducted at different temperatures (700, 850, and 1000°C) under a nitrogen gas atmosphere with a heating rate of 2.5 deg/min and a holding time of 5 h. Physicochemical characterizations have indicated that nickel acetate is entirely reduced to nickel metal during the sintering process, and molybdenum has bonded with carbon to produce molybdenum carbide. At the same time, the used polymer has been pyrolyzed to produce a carbon nanofiber matrix embedding formed inorganic nanoparticles. Electrochemical measurements concluded that molybdenum content and calcination temperature should be controlled to maximize the electrocatalytic activity of the proposed catalyst. Typically, the oxidation peak current density was 28.5, 28.8, 51.5, 128.3, 25.6, and 3 mA/cm^2^ for nanofibers prepared from an electrospun solution containing 0, 5, 10, 15, 25, and 35 wt% molybdenum carbide, respectively. Moreover, it was observed that increasing the calcination temperature distinctly improves the electrocatalytic activity. Kinetic studies have indicated that the reaction order is close to zero with a reaction temperature-dependent value. Moreover, it was detected that the electrooxidation reaction of ethanol over the proposed nanofiber composite follows the Arrhenius equation. The determined activation energy is 33 kJ/mol, which indicates good catalytic activity for the introduced nanofibers. Through the application of a set of visualization-based tools and the general linear model (GLM), the optimal conditions that generate the highest current density were identified. The computations unveiled that the optimal parameter settings are as follows: Mo content at 15 wt.%, methanol concentration of 1.55 M, and reaction temperature of 59°C.

## 1. Introduction

The quest for efficient and sustainable energy sources has intensified in recent years, driving extensive research into advanced materials for electrocatalysis, particularly for ethanol electrooxidation. Ethanol holds immense promise as a renewable fuel due to its high energy density, low toxicity, and compatibility with existing infrastructure [1–4]. However, the lack of cost-effective and highly active electrocatalysts for the ethanol oxidation reaction (EOR) [5, 6] hinders the widespread adoption of ethanol fuel cells. In this regard, developing novel electrocatalytic materials capable of promoting the EOR with high efficiency and stability remains a critical challenge.

Enhancing anode performance can be attained through various approaches, including surface modification, the utilization of bifunctional catalysts, optimized electrode design, and careful management of operational parameters [7]. However, among these strategies, the paramount factors contributing to heightened electrode electroactivity are the choice of electrode material and its design. Thus, exploring anode electrocatalysts that bolster EOR activity and mitigate the adverse effects of CO poisoning seven becomes imperative. Notably, although pure platinum remains the most proficient electrocatalyst for EOR, its extensive implementation is impeded by its exorbitant cost and susceptibility to CO intermediate-induced poisoning [8]. In response, alloying platinum with other metals such as Ru, Ir, Au, Ag, Pd, Fe, and Co has been proposed as an efficacious means to enhance electrocatalytic activity [9–13]. Nevertheless, the challenge posed by cost considerations persists.

The utilization of advanced catalyst supports is essential for optimizing electrocatalytic performance. Carbon nanofibers (CNFs) have garnered attention as an exceptional electrocatalyst support material. Their high surface area, excellent electrical conductivity, and tunable pore structure make CNFs ideal for enhancing catalyst dispersion, stability, and overall performance in ethanol oxidation [14, 15]. Examples of the utility of CNFs include their use in improving the catalytic activity of transition metals and their alloys for various electrochemical reactions [14, 16]. Additionally, their compatibility with different catalytic species makes CNFs a versatile substrate for promoting catalytic activity [17].

Due to their unique electronic structure and surface properties, transition metal carbides have garnered considerable attention as potential catalysts for various electrochemical reactions. Nickel (Ni), a widely employed non-precious electrocatalyst, has demonstrated proficiency in ethanol electrooxidation. However, to enhance the catalytic activity of Ni towards ethanol oxidation, incorporating co-catalysts becomes imperative [18, 19].

Distinguished by its unique attributes and exceptional catalytic prowess across various reactions, molybdenum carbide emerges as a standout transition metal carbide. Its capabilities encompass diverse processes, including the selective isomerization of hydrocarbons [20], ammonia synthesis [21], hydrofining [22], hydrodeoxygenation (HDO) of phenols [23], water-gas shift [24], hydrogen evolution reaction [25], hydrodesulfurization [26], and syngas to lower alcohol [27]. When employed as an electrocatalyst, this compound has demonstrated commendable electrocatalytic performance in the selective oxidation of methanol and hydrogen electrooxidation reactions [28, 29]. Moreover, molybdenum carbide has been harnessed as a co-catalyst to enhance the electrocatalytic activity of initial catalysts [30, 31]. Although this effective co-catalyst has been exploited to improve the Pt electrocatalytic performance toward EOR [31]. To the best of our knowledge, combining molybdenum carbide with nickel to augment electrocatalytic activity in EOR remains unexplored.

This manuscript introduces a novel Ni-Mo-C-graphite nanofiber composite as an efficient electrocatalyst for ethanol electrooxidation. The synthesis of this composite involves the calcination of electrospun nanofibers composed of nickel acetate tetrahydrate, molybdenum chloride, and polyvinyl alcohol at different temperatures under a nitrogen gas atmosphere. Through meticulous physicochemical characterizations, we elucidate the structural evolution of the composite during the sintering process, revealing the formation of nickel metal and molybdenum carbide embedded within a carbon nanofiber matrix.

A set of visualization-based tools has been invoked to target the conceptual evaluation of interdependences among the factors controlling the process to mine the optimum conditions yielding the maximum current density and, hence, the amount of green hydrogen produced. The general linear model (GLM) fits the data since the response parameter, PCD, is continuous, including four covariates and their interaction and polynomial terms. This approach aids in optimizing experimental parameters, such as catalyst composition, loading, and operating conditions, to achieve optimal catalytic performance. The results are promising as the proposed catalyst showed a significant electrocatalytic performance toward ethanol electrooxidation.

## 2. Materials, experiments, and methods

### 2.1 Materials

All utilized chemicals, purchased from Sigma Aldrich, Seoul, South Korea, were of the highest possible purity (≥ 99%). They were used without further processing or purifications. Nickel acetate tetrahydrates (Ni(Ac)_2_, Ni(CH_3_COO)_2_.4H_2_O), Molybdenum chloride (MoCl_2_), and Polyvinyl alcohol (PVA, molecular weight (Mwt) ≈ 65,000 g/mole) were the main employed ingredients. The PAV polymer was used to form the electrospun solution. Deionized water with a resistivity higher than 17 MΩ was employed as the solvent.

### 2.2. Catalyst-composite fabrication and the experimental methods

**Fig 1:**
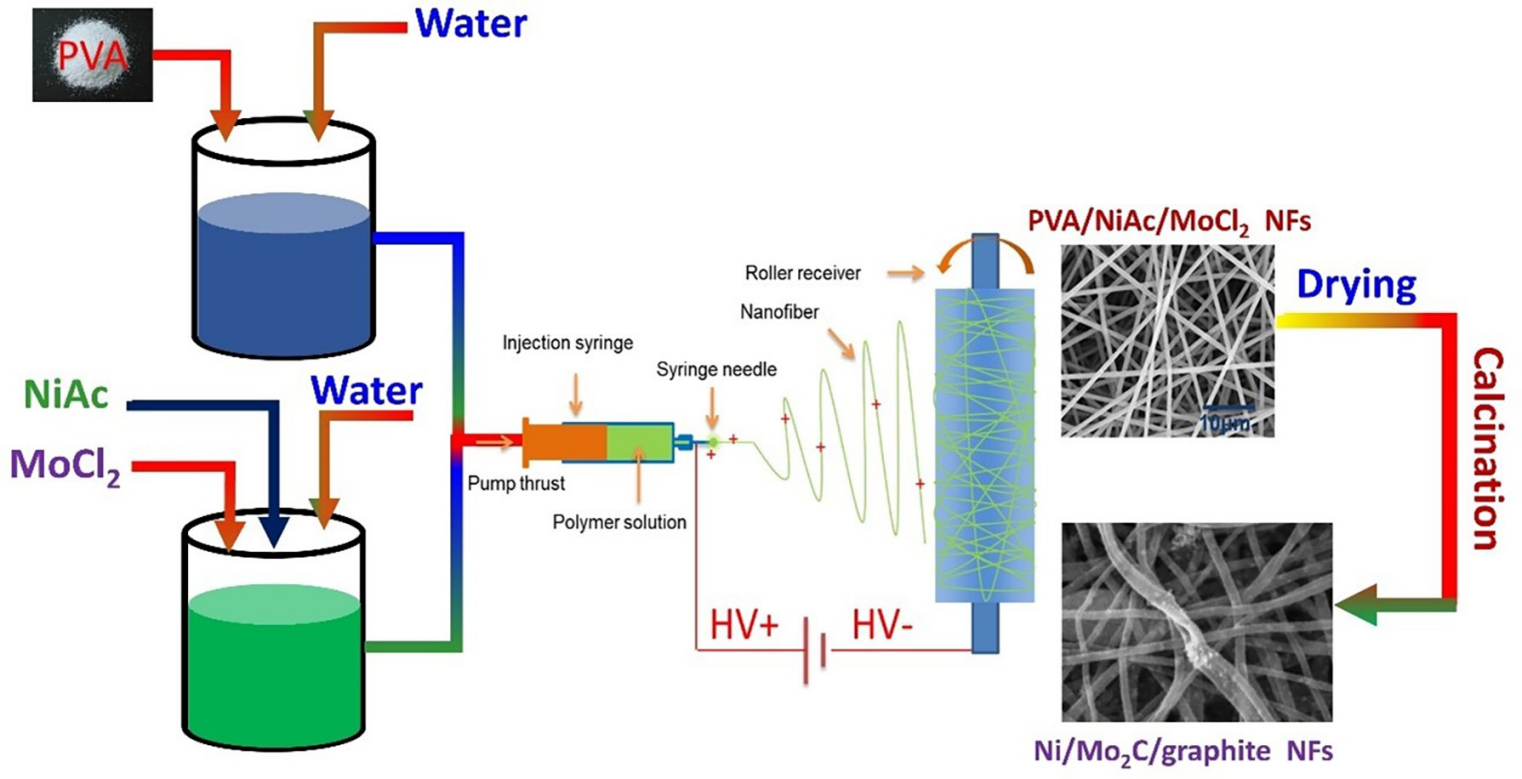
Graphical explanation for Ni/Mo_2_C/graphite NFs synthesis procedure.

The nickel acetate/PVA aqueous stock was ready by mixing three-to-one fractions of 10wt% PVA and 20wt% nickel acetate aqueous solutions, respectively. First, PVA aqueous solution was prepared as follows: 10 g of the polymer granules were gradually dispersed in 90 mL of deionized water agitated vigorously, and the stirring continued overnight at 50°C in a controlled water bath. The electrospun sol-gel was prepared by mixing 15 g from the prepared polymer solution with 5 mL of deionized water containing 1 g of Ni(Ac)_2_. The stirring of the resultant admixture was continued for 5 hrs. at 50°C. Afterward, preparation of a series of sub-stock solutions containing 0, 5, 10, 15, 25, and 35 wt.% of MoCl_2_ relative to Ni(Ac)_2_ was accomplished by dissolving predetermined amounts of MoCl_2_ in a tiny amount of deionized water and finally added to the respective volume of the nickel acetate/polymer solution. A gap of 15 cm between the syringe and the rotating drum collector was adjusted in the electrospinning machine operated at a voltage of 20 kV. The vacuum-dried electrospun mats were pyrolyzed (i.e., calcined under vacuum) for 5 h at preselected temperatures of 700, 850, and 1000°C. The experimental procedure is graphically illustrated in Fig 1.

### 2.3. Characterizations

The produced materials underwent comprehensive characterization at the Central Laboratory for Microanalysis and Nanotechnology, Minia University. The morphology of the fabricated nanofibers was examined using scanning electron microscopy (SEM) and field-emission scanning electron microscopy (FESEM), employing a Hitachi S-7400 scanning electron microscope from Japan. For a detailed analysis of the chemical composition of the nanostructures, we utilized an X-ray diffractometer from Rigaku, Tokyo, Japan.

In addition, we conducted a thorough transmission electron microscopy (TEM) analysis of the materials using a Phillips CM12 TEM instrument equipped with an elemental mapping tool. A copper grid was immersed in the slurry and air-dried to prepare the samples for TEM analysis before being loaded into the instrument. Before loading, the samples were sonicated in pure ethanol for 10 min to ensure uniform dispersion.

A potentiostat (VersaStat 4, Ametek Scientific Instrument, Berwyn, PA, USA) in a three-electrode cell setup was employed to evaluate the electrochemical performance of the functional materials. This configuration consisted of a working electrode, an Ag/AgCl electrode used as the reference electrode, and a Pt wire serving as the counter electrode. The working electrode was prepared by applying a 15 μL aliquot of the catalyst ink evenly onto the active surface of a glassy carbon electrode. The electrode was then dried for 30 min at 80°C. The catalyst ink formulation was prepared by dispersing 2 mg of the active component in a mixture containing 20 μL of Nafion solution and 400 μL of isopropanol. All measurements were conducted in a 1.0 M KOH solution. Cyclic voltammetry measurements were performed for at least 3 cycles, the last cycle was displayed in the introduced data.

### 2.4 Statistical manipulations and data analysis

We utilized various visualization tools focused on conceptually assessing the relationships among the factors that govern the process. The goal was to identify the optimal conditions that would result in the highest current density, consequently leading to an increased production of green hydrogen. We employed the 3D surface plots, bubble diagrams, and contour plots presentation methods as they are readily readable and interpretable curves. Moreover, the Spearman Rho correlations matrix has been exploited. The covariates’ main effects, interaction plots, and dynamic response optimization plots have been constructed based on the general linear model analysis.

## 3. Results and discussion

### 3.1 Synthesis reactions mechanism

**Fig 2:**
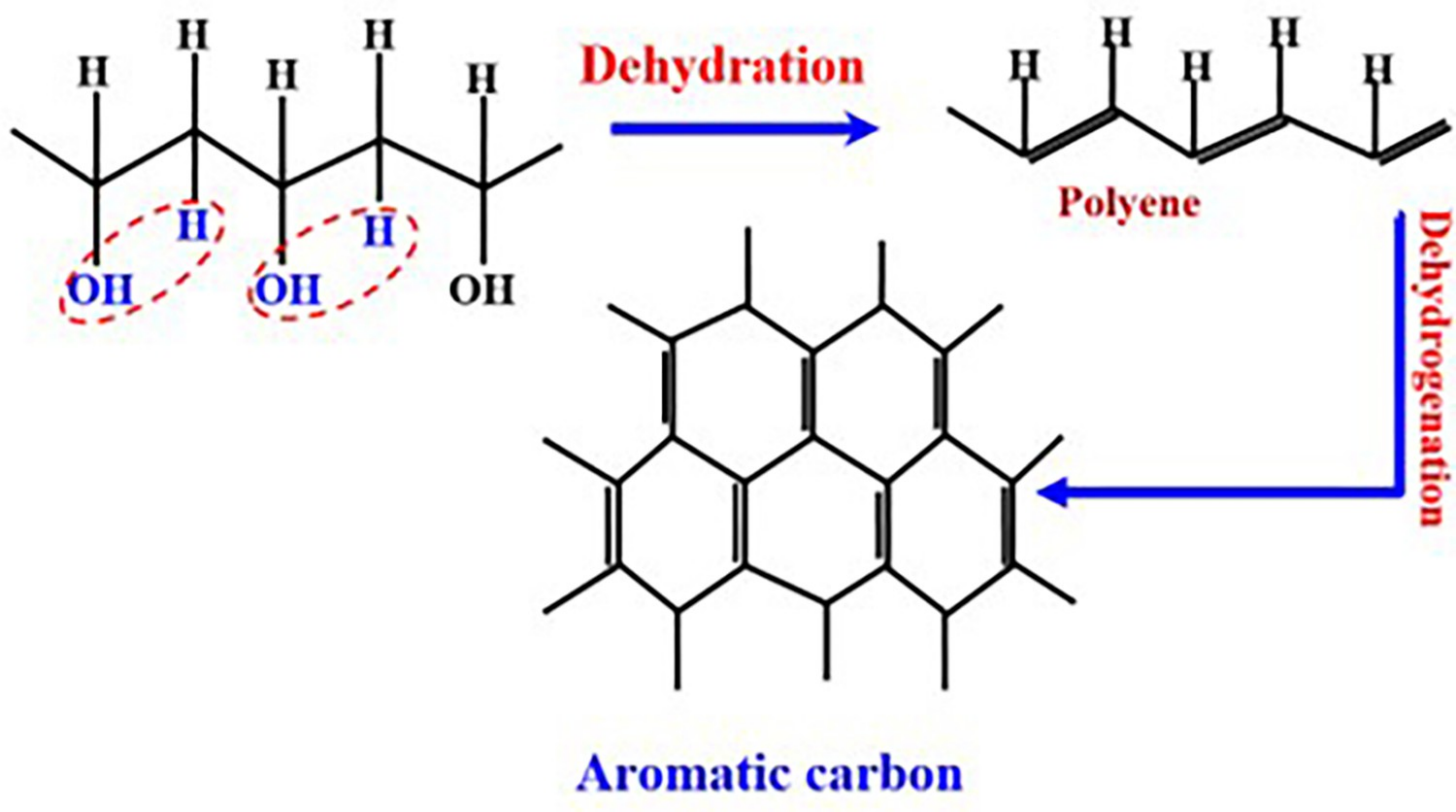
Schematic diagram for the expected optimum destructive of the PVA [32, 33].

PVA possesses a notably higher carbon content than other vinyl polymers; nevertheless, its utilization in crafting carbon nanofibers is limited due to challenges stemming from its low-temperature melting and subsequent decomposition into volatile, low-molecular-weight compounds. Two primary strategies have been adopted to address this issue. The initial method entails pre-treatment before the carbonization process, while the second method entails using specific catalysts during the heat treatment to boost the graphitization process. Both approaches involve iteratively altering the PVA straight chain, resulting in compounds with higher melting points that notably expedite graphitization [32]. A schematic representation in Fig 2 highlights the optimal destructive disintegration of PVA to achieve maximal yield.

Techniques such as dehydration and dehydrogenation have been employed to extract aromatic carbon from the PVA straight chain, as depicted. The selection of nickel acetate as a precursor serves dual purposes. Under inert atmospheres, this precursor decomposes entirely to zero-valent nickel rather than forming the anticipated nickel oxides. This decomposition releases reducing gases—carbon monoxide and hydrogen—leading to pure metal generation, as numerous researchers demonstrated [34, 35]. The following equations can describe the formation of pure nickel.


$$Ni{\left(CH_{3}COO\right)}_{2}\cdot 4H_{2}O\to 0.86Ni{\left(CH_{3}COO\right)}_{2}\cdot 0.14Ni{\left(OH\right)}_{2}+0.28CH_{3}COOH+3.72H_{2}O$$ (1)

$$3(0.86\;Ni{\left(CH_{3}COO\right)}_{2}\cdot 0.14Ni{\left(OH\right)}_{2})+0.28H_{2}O\to 3NiCO_{3}+2.44CH_{3}COCH_{3}+1.12H_{2}$$ (2)

$$NiCO_{3}\to NiO+CO_{2}$$ (3)

$$NiO+CO/H_{2}\to Ni+CO_{2}/H_{2}O$$ (4)

**Fig 3:**
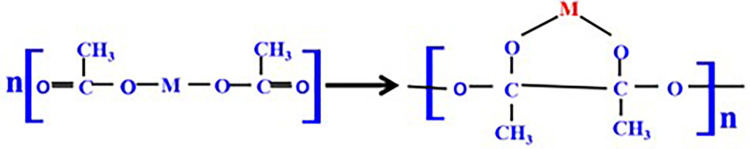
The polycondensation reaction of Nickel acetate.

Also, nickel acetate exhibits a polycondensation propensity, effectively preserving the nanofibrous morphology during calcination. This polycondensation reaction can be expressed as shown in Fig 3: [32].

In this context, "M" represents the nickel atom. As a result, the selected precursors form a cohesive gel with the polymer during the electrospinning process, simplifying the efficient production of nanofibers. Also, pure nickel formation enhances the polymer’s thermal stability during calcination.

### 3.2 Catalysts morphology and composition

**Fig 4:**
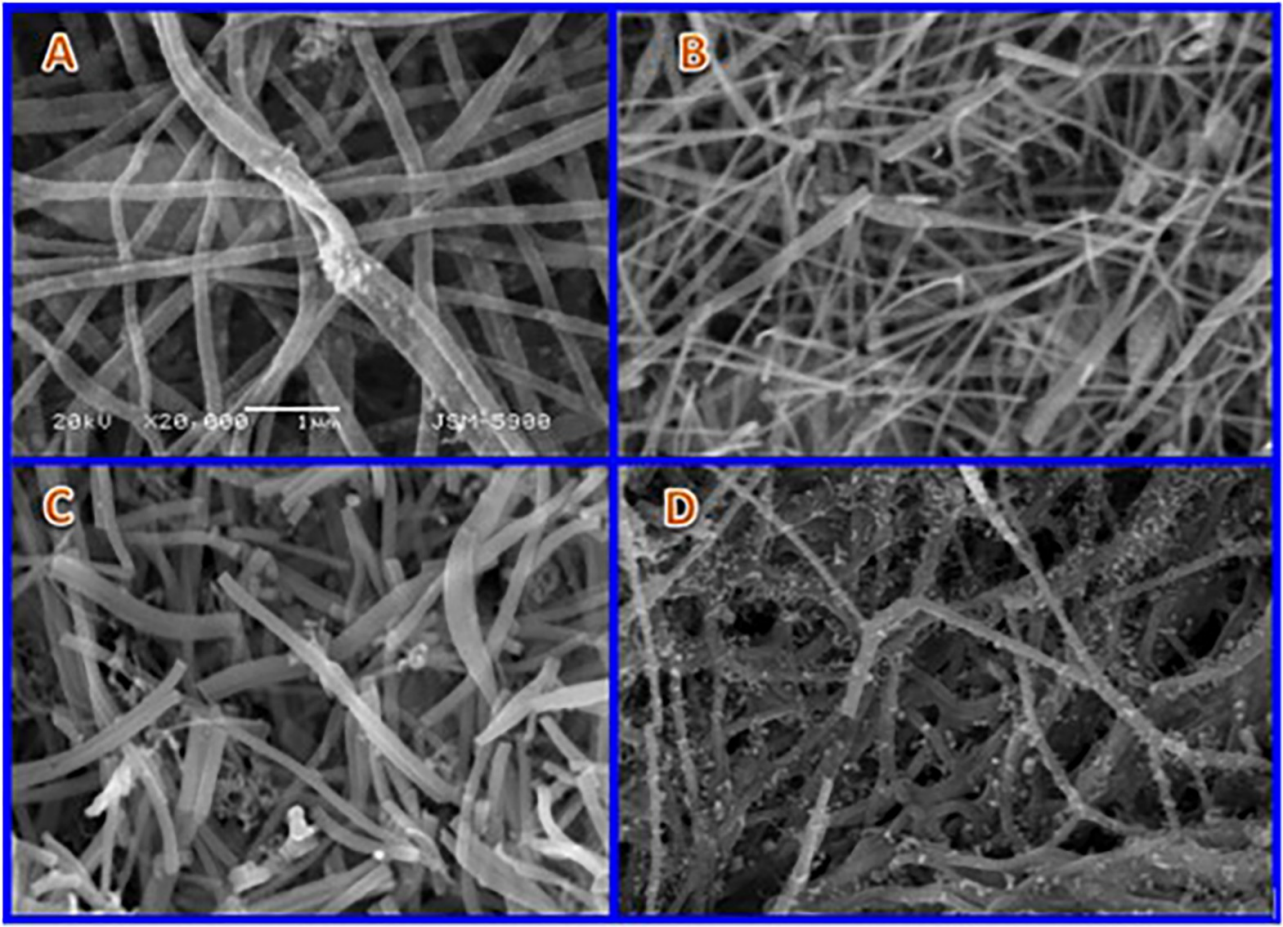
SEM images of the fabricated Mo_2_C-Ni-C composite nanofibers, synthesized through calcination at 850°C. The nanofibers originate from the initial solution with varying molybdenum chloride concentrations: 0 (A), 10 (B), 15 (C), and 35 (D). The scale bar matches 1 μm.

SEM images presented in Fig 4 depict randomly chosen samples after the heat treatment phase at 850°C. These samples include those prepared with zero (Fig 4A), 10 (Fig 4B), 15 (Fig 4C), and 35 (Fig 4D) wt% MoCl_2_. Analysis of these panels shows that the incorporation of molybdenum chloride, within the stipulated range of this investigation, does not impede the electrospinning feasibility of the prepared solutions. Furthermore, the initial morphology exhibits thermal stability; subjecting the electrospun nanofibers to the high-temperature treatment process does not compromise the integrity of their nanofibrous structure. The nanoparticles observed adhering to the primary nanofibers in cases of elevated molybdenum precursor content (Fig 3D) can be ascribed to the adverse impact of molybdenum chloride on the polycondensation process of the acetate salt. This influence gives rise to the formation of beads during the electrospinning procedure, which subsequently transforms into the observed minute nanoparticles. Consequently, it can be reasonably deduced that the nanoparticles adhering to the nanofibers share a similar composition to the primary nanofibers.

**Fig 5:**
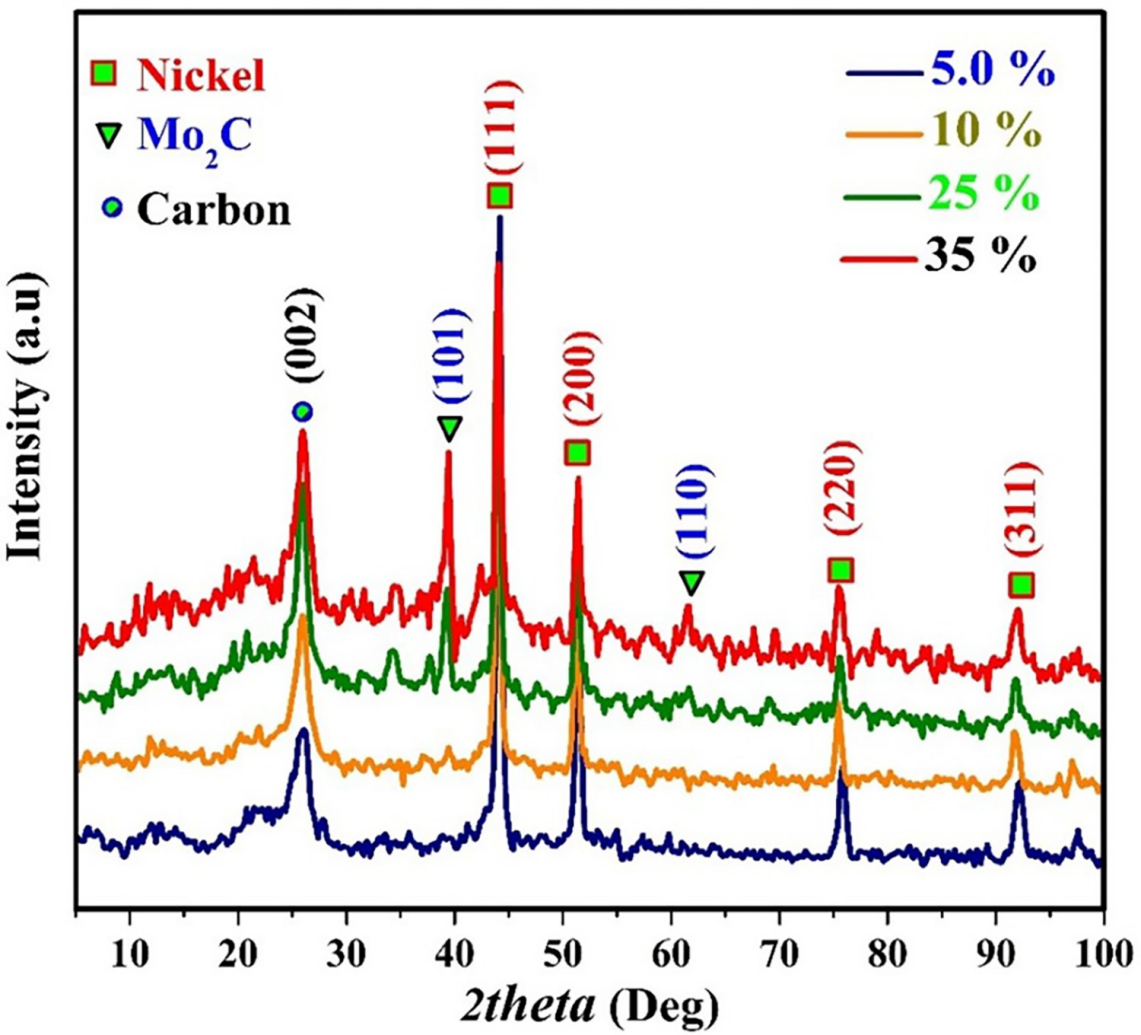
X-ray diffraction (XRD) patterns for the produced powders from sintering nanofiber mats containing different molybdenum chloride content at 850°C.

X-ray diffraction analysis is a well-established and reliable method for determining the chemical composition of crystalline materials. In this study, we conducted XRD pattern analysis for the synthesized products, and Fig 5 displays representative XRD patterns for selected samples. These findings support the previously discussed pathway of thermal decomposition for nickel acetate. As shown, distinct peaks attributed to Ni were clearly observed in all formulations. These conclusions are further confirmed by the prominent peaks at 2θ values of 44.30°, 51.55°, 76.05°, and 92.55°, corresponding to crystal planes identified by Miller indices (111), (200), (220), and (311), respectively (JCDPS# 04–0850). Furthermore, a relatively broad peak at around ~26.3° with a d-spacing of 3.37 Å confirms the presence of graphite-like carbon (specifically graphite-2H, d (002), JCPDS#; 41–1487), indicating the successful graphitization of the PVA component. Molybdenum formed a chemical bond with a portion of the resulting carbon, resulting in the formation of thermally stable molybdenum carbide (Mo_2_C) characterized by the lowest oxidation state of Mo (2). This phenomenon can be attributed to the release of reducing gases during the calcination process [36]. The indexed Mo_2_C peaks (JCPDS; # 35–0787) were confirmed and exhibited higher intensity with an increase in the molybdenum precursor content in the samples.

**Fig 6:**
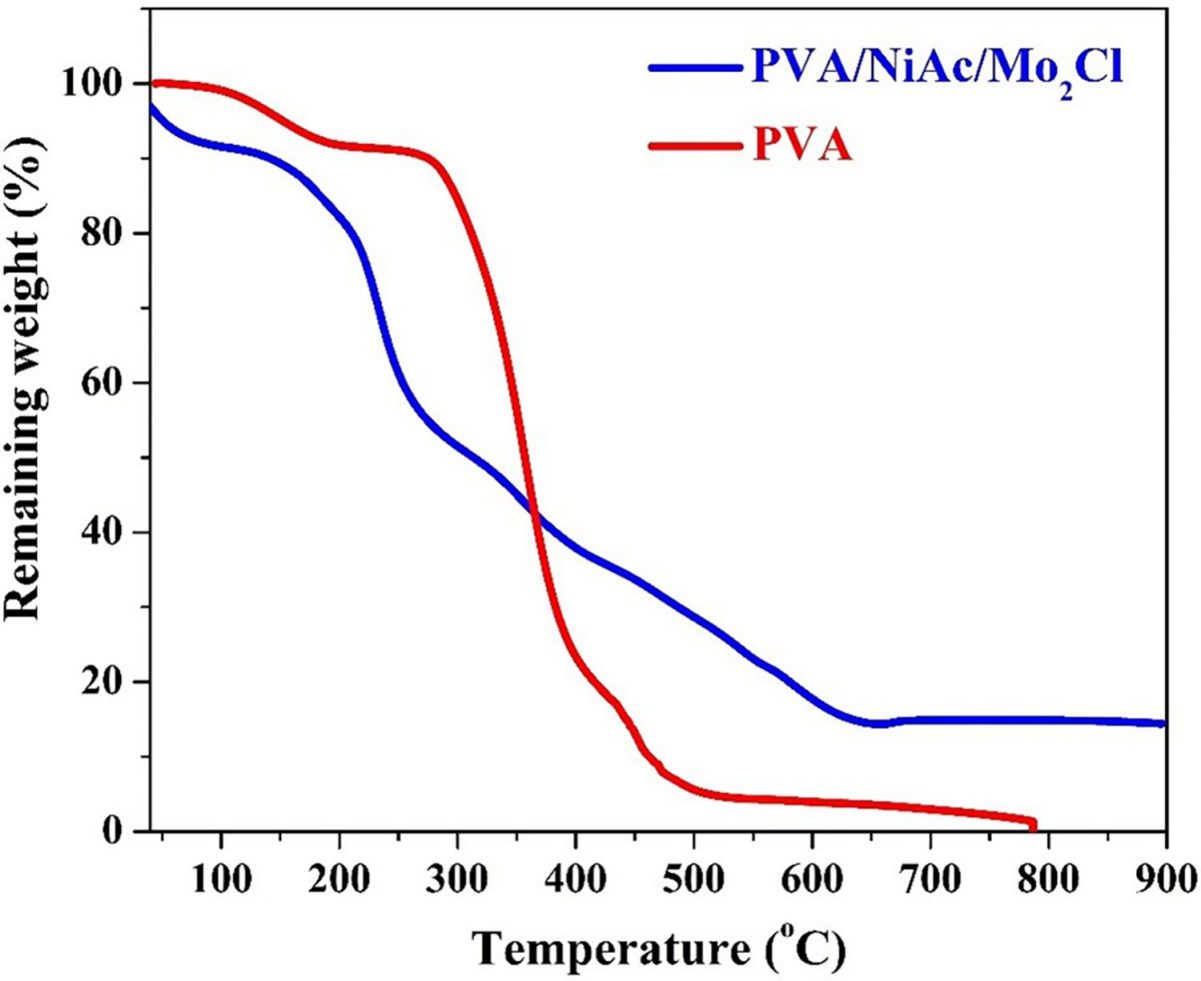
Thermal gravimetric analysis for PVA and PVA/NiAc/Mo_2_C composite.

The Thermogravimetric Analysis (TGA) results, depicted in Fig 6, offer valuable insights into the weight loss behavior during the carbonization process of the PVA/NiAc/MoCl_2_ nanofiber composite. TGA is a powerful technique for studying the thermal decomposition and stability of materials, particularly during heat treatment processes. As shown in the figure, pristine PVA was completely decomposed, a known thermal characteristic of this polymer [37]. For the composite polymeric nanofibers, the initial stage of the TGA curve reveals a sharp weight loss corresponding to the decomposition of the polymeric component (PVA). The sharp decline begins around 200°C, consistent with the decomposition temperature of PVA. Later, there is a gradual decrease in the weight which can be assigned to the decomposition of the acetate ion, Eqs 1 to 4 [34, 35]. After these weight losses, the TGA curve levels off, indicating the formation of a stable residue. This stable residue constitutes approximately 15% of the original mass of the polymeric nanofiber composite. Notably, from 650°C until the maximum temperature utilized (900°C), no additional weight loss is observed. This plateau suggests the formation of thermally stable products and the absence of further decomposition or loss of volatile components.

The observed stable residue corresponds to the inorganic components, primarily nickel and molybdenum carbide as can be concluded from the XRD results. These TGA results contribute to our understanding of the thermal behavior of the PVA/NiAc/MoCl_2_ nanofiber composite during the carbonization process. The stability of the residue supports the retention of the inorganic components, emphasizing the suitability of the carbonization conditions for preserving the desired Ni and Mo content in the final nanofiber structure.

**Fig 7:**
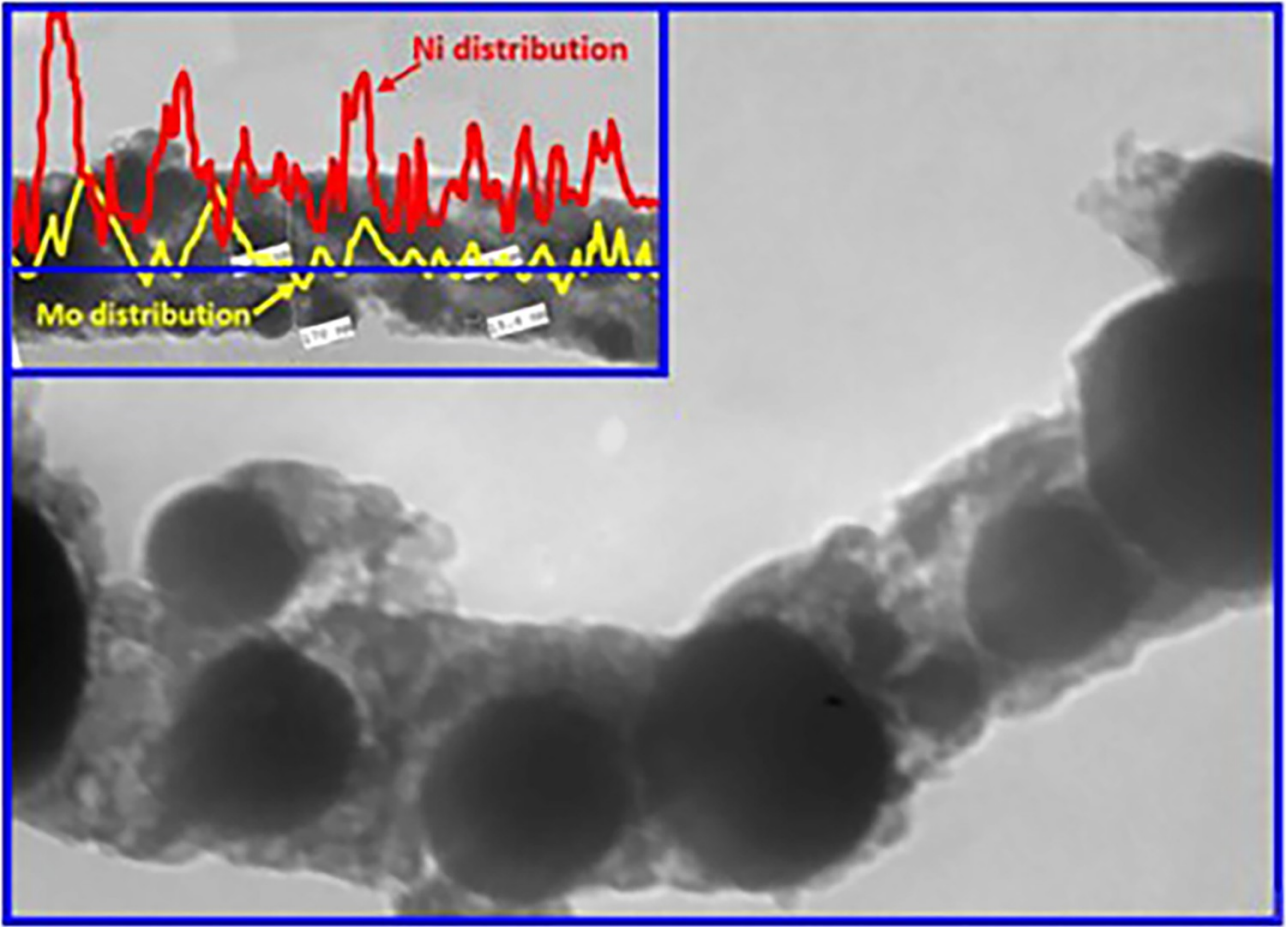
Transmission electron microscopy (TEM) depicts the Mo_2_C/Ni/graphite composite nanofibers formed by calcinating a 10% Mo-containing electrospun solution at 850°C. Linear elemental mapping highlighting the distribution of Ni and Mo along randomly chosen regions is indicated in the inset.

Utilizing Transmission Electron Microscopy (TEM) as an established analytical tool allows for examining the internal structure of nanostructures. Applying electron beams lead to strong reflections, causing crystalline materials to appear as dark regions in TEM imaging. The TEM image in Fig 7, depicting the 10 wt.% nanofibers subjected to 850°C calcination, unequivocally confirms the presence of an internal structure within the generated nanofibers. Notably, graphite is observable within the grey matrix during TEM investigation, suggesting that the dark areas correspond to equivalent inorganic constituents within the nanofibers. Furthermore, a comprehensive elemental mapping analysis was conducted along a randomly chosen line. The results, as illustrated in the inset of Fig 7, reveal distinct distributions of nickel and molybdenum. This observation leads to the conclusion that these two metals do not form an alloy structure. This finding is consistent with the XRD results, which indicated the presence of molybdenum in the form of molybdenum carbide. Consequently, the combined physicochemical characterizations collectively validate the effectiveness of the proposed preparation method in producing carbon nanofibers incorporating Mo_2_C/Ni nanoparticles as the final product.

### 3.3 Electrochemical measurements

#### 3.3.1 Effect of molybdenum incorporation

Considering the significantly high melting points of nickel (1455°C) and molybdenum carbide (2687°C), the expectation of their evaporation during the preparation process is negligible. Hence, the proportion of the metal carbide co-catalyst in the composite can be inferred to be 7.12%, 13.30%, 18.71%, 27.73%, and 34.94% by weight relative to the primary catalyst. This relationship applies to the nanofibers produced from electrospun solutions containing molybdenum chloride salt at concentrations of 5%, 10%, 15%, 25%, and 35%, respectively.

**Fig 8:**
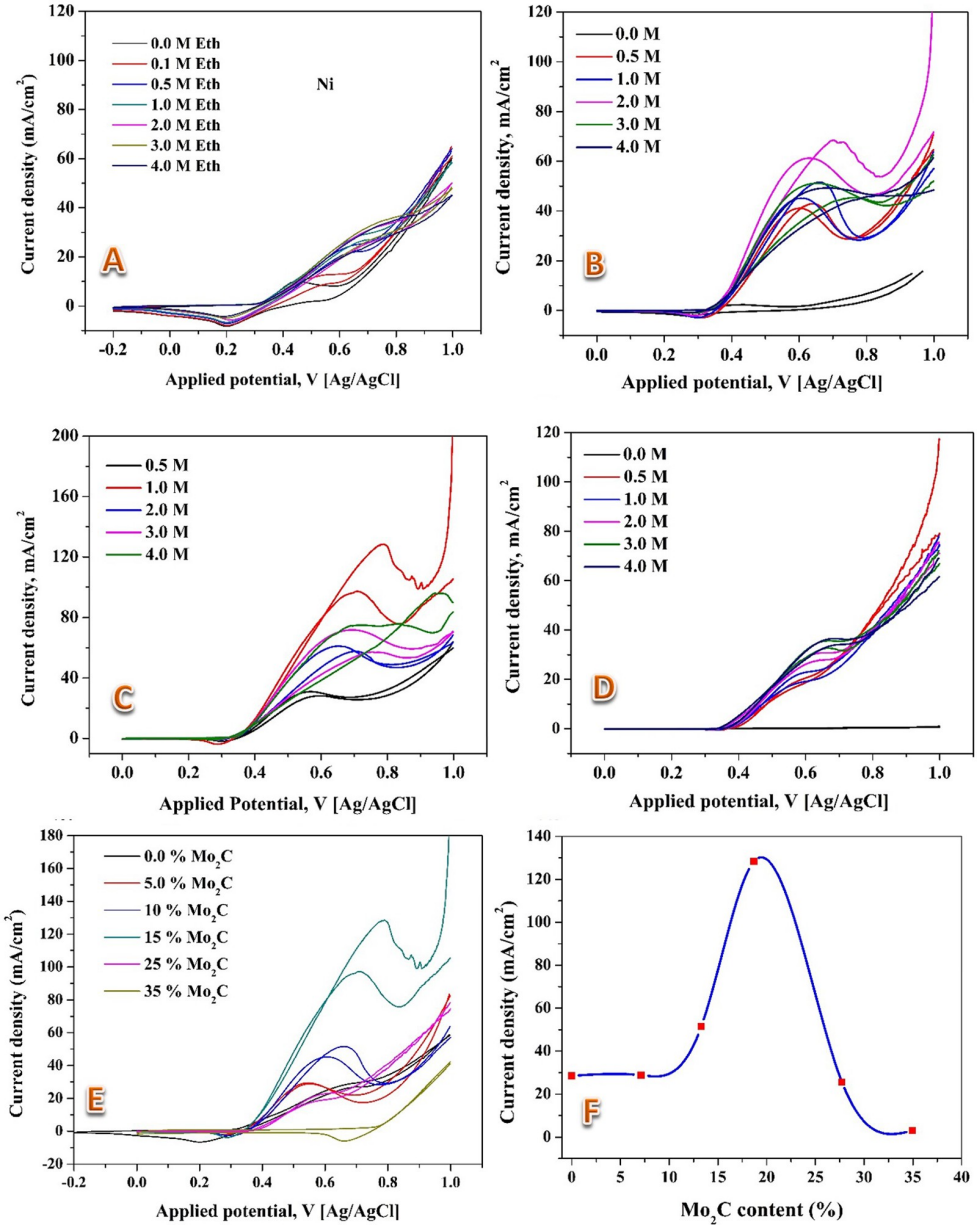
Electrocatalytic Activity Trends of Nanofibrous Catalyst with Varied Molybdenum Carbide Content: A) 0.0% Mo_2_C, B) 13.3% Mo_2_C, C) 18.7% Mo_2_C, D) 27.7% Mo_2_C. Panels E and F display the electrocatalytic activity of all prepared nanofiber electrodes at 1.0 M ethanol and the maximum power density at 1.0 M ethanol, respectively. The measurements have been conducted at 50 mV/s scan rate and 25°C.

Cyclic voltammetry measurements were carried out to investigate the effect of molybdenum carbide content on the catalytic activity of the introduced nanofibrous catalyst prepared at 850°C, the results are introduced in Fig 8. Typically, the proposed composite nanofibers have been prepared using different contents of the suggested co-catalyst, then the cyclic voltammetry measurements were performed at different ethanol concentration with an applied voltage window -0.2 ~ 1.0 V at scan rate 0.05 V/s and 25°C reaction temperature. Beginning with the Mo-free electrocatalyst (Fig 8A), moderate electrocatalytic activity is evident, with an oxidation peak current density of approximately 28 mA/cm^2^. Notably, an incremental surge in current density is observed upon elevating the ethanol concentration from 0.1 M to 0.5 M. This limited responsiveness is likely attributed to saturation effects stemming from higher ethanol concentrations. With the addition of 5% molybdenum chloride in the initial electrospun solution, minimal alteration in electrocatalytic activity is noted (data not shown). However, a more pronounced enhancement emerges with 10% molybdenum chloride (Fig 8B), resulting in an oxidation peak current density of 69 mA/cm^2^ at the optimal ethanol concentration of 2.0 M. This configuration showcases current densities of 43 and 51 mA/cm^2^ for ethanol concentrations of 1 and 3 M, respectively. A more substantial improvement is apparent with 15% molybdenum chloride (Fig 8C), marked by a sharp increase in oxidation peak current density, reaching 128 mA/cm^2^ at the optimal ethanol concentration of 1.0 M–a nearly five-fold enhancement compared to the Mo-free electrocatalyst.

Interestingly, the optimal ethanol concentration for this configuration aligns with the maximum catalytic performance observed. Beyond this point, however, an inverted trend becomes apparent. With 25% molybdenum chloride (Fig 8D), the electrocatalytic activity diminishes, evidenced by a maximum current density of 37 mA/cm^2^ at an ethanol concentration of 3.0 M. For reference, Fig 8E displays the influence of molybdenum content specifically for a 1.0 M ethanol solution. A Gaussian distribution curve in Fig 8F encapsulates the relationship between co-catalyst content and oxidation peak current density for a 1.0 M ethanol solution, with a peak at 15% Mo. This intricate trend suggests that adding molybdenum carbide initially augments the electrocatalytic activity through synergistic effects. However, an excessive increase in molybdenum content could lead to unfavorable interactions, subsequently diminishing catalytic performance. The precise underlying mechanisms governing this intricate behavior warrant further investigation, potentially involving phenomena such as changes in active site density, electronic interactions, and mass transport limitations. Overall, these results provide crucial insights for optimizing the composition of nanofibrous catalysts for enhanced electrocatalytic activity.

#### 3.3.2 Influence of nano-structural morphology

The electron transfer resistance, a critical determinant of electrocatalyst performance, is significantly influenced by the catalyst’s morphology. This relationship becomes particularly evident when considering the axial ratio, representing a nanostructure’s length ratio to its diameter or width. The axial ratio significantly influences the catalytic performance of the functional material. Generally, elongated nanomaterials exhibit superior electrocatalytic activity compared to their spherical or shorter counterparts. This enhanced performance can be attributed to various factors. Firstly, elongated nanomaterials offer, per unit volume, a greater surface area, providing new active sites sharing in the catalytic reactions. Secondly, their long size facilitates the diffusion of used reactants and the produced materials, resulting in faster reaction rates. The elongated structures can also encourage forming new crystal facets that exhibit heightened electrocatalytic performance [38].

Furthermore, electrocatalysts encounter interfacial and inherent ohmic resistances, which hinder the transfer of electrons through the catalyst’s interface layer. Consequently, minimizing interfacial resistance may substantially enhance electron transfer, improving electrocatalytic activity. The distinctive long axial ratio intrinsic to the nanofibrous morphology creates direct pathways to the collector, markedly decreasing the formed resistance between phases in contrast to particulate shapes. In nanoparticle configurations, electrons must navigate multiple contact sites in a zigzag manner, resulting in notable interfacial resistances. Conversely, the elongated axial ratio facilitates direct electron pathways to the collector, decreasing the resistance collector [39].

**Fig 9:**
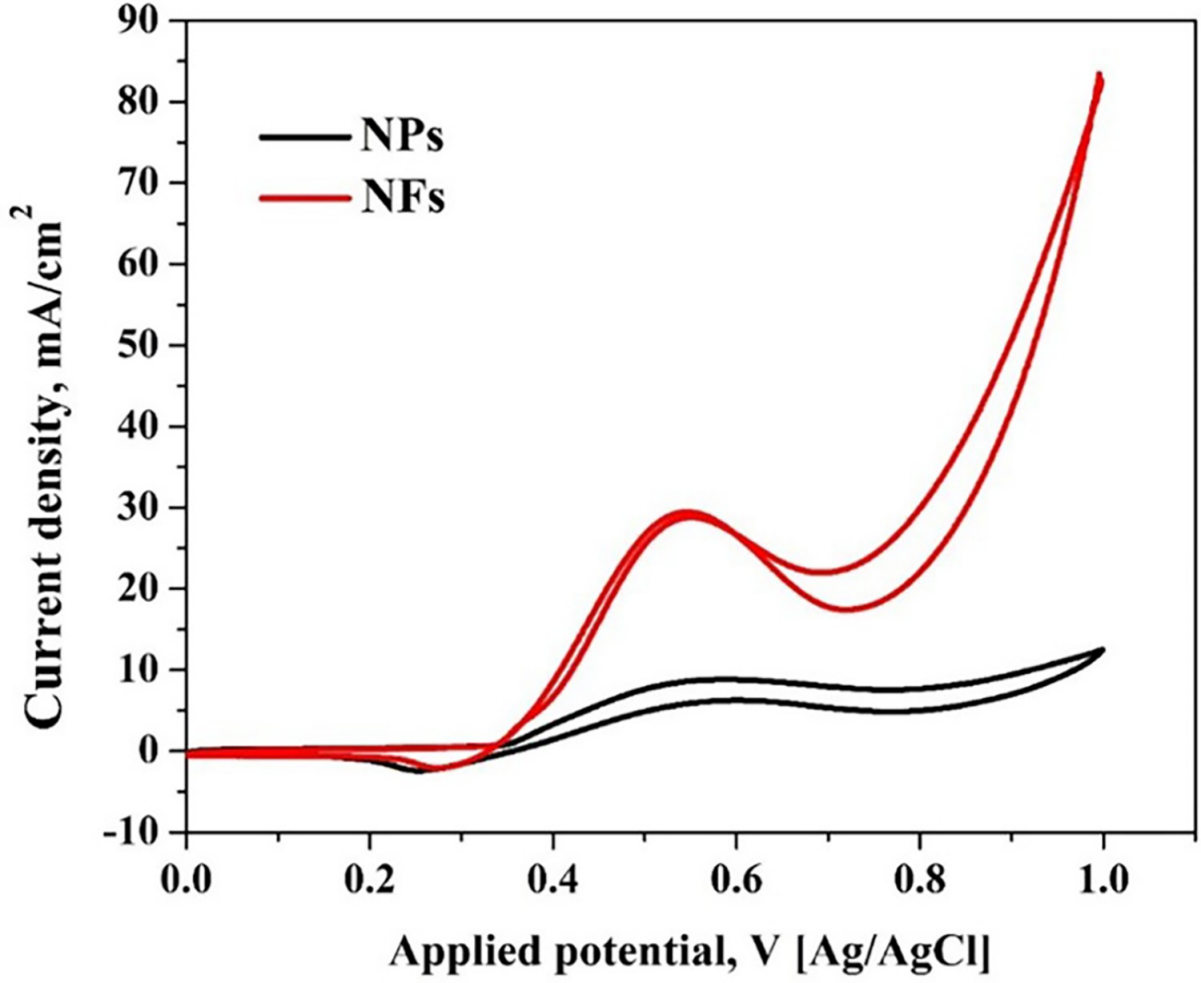
Effect of structural morphology on the EOR recorded for 5% Mo/Ni nanofiber composite composition calcined at 850°C, 1 M ethanol, and a scan rate of 50 mV/s.

This concept is systematically validated by generating nanoparticles using the same sol-gel employed for electrospinning, followed by calcination at identical conditions, specifically 5 wt.% MoCl_2_ at 850°C. As anticipated, the two shapes manifest a substantial difference in catalytic performances, affirming the advantages of the fibrous shape, as illustrated in Fig 9.

#### 3.3.3 Effect of the calcination temperature

The calcination temperature wields substantial influence over the attributes of electrocatalysts, which is critical in enhancing their catalytic activity, stability, and selectivity. Elevated calcination temperatures typically yield electrocatalysts with heightened crystallinity and reduced surface area. This is due to the propensity of calcination to induce sintering or fusion among particles, resulting in a decrease in total surface area [40]. However, this phenomenon also bolsters the catalyst’s stability and longevity. Additionally, calcination can induce alterations in the electrocatalyst chemical composition. At elevated temperatures, specific chemical reactions can be achieved, subsequently modifying the composition of the catalyst [41].

**Fig 10:**
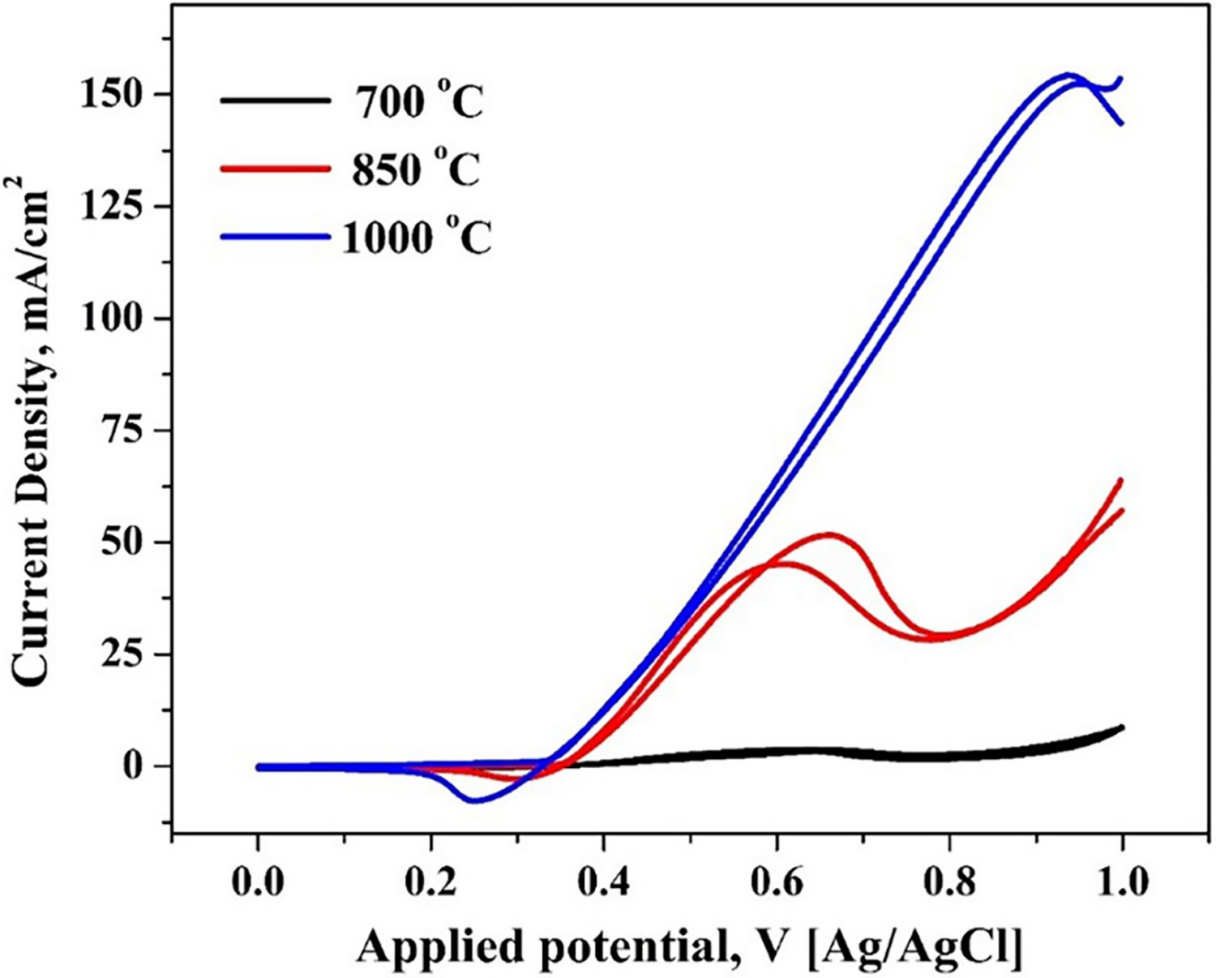
Effect of variation in sintering temperature on the catalytic activity 10% Ni-Mo-incorporated CNF, 1 M ethanol & 0.05 V/s.

For instance, the calcination of carbon-based electrocatalysts can eliminate oxygen-containing functional groups, increasing their catalytic performance. Conversely, sintering of Pt-based electrocatalysts at elevated temperatures might trigger Pt NPs’ agglomeration, leading to larger particles that may, in turn, diminish their electrocatalytic efficiency [42, 43]. Conversely, sintering may also foster the creation of new active sites on the surface of the electrocatalyst, enhancing the overall performance. Thus, selecting an appropriate calcination temperature is crucial in electrocatalyst development. The optimal temperature hinges on the catalyst’s distinct characteristics and the required electrochemical activity. In Fig 10, cyclic voltammetry curves of 10 wt.% anodes prepared at various temperatures during 1.0 M ethanol exposure reveal the impact of preparing temperature on the investigated anode electroactivity. Notably, ethanol oxidation peak is clearly observed at low applied voltage for the sample prepared at 850°C, producing pronounced current and a well-defined ethanol oxidation peak attributed to the excellent crystallinity achieved at such elevated temperatures. Although, for the 700°C nanofibers, the oxidation peak was observed at almost similar applied potential of the 850°C formulation (0.66 V), the corresponding current density is trivial (3.7 mA/cm^2^) compared to 51.6 mA/cm^2^ obtained in the case of 850°C nanofibers. On the other hand, increasing the calcination temperature to 1000°C strongly increased the oxidation peak current density to 154 mA/cm^2^; regrettably, the corresponding required potential increased to 0.95 V, which is economically unpreferred. Accordingly, it can be claimed that 850°C is the optimum calcination temperature.

#### 3.3.4 Kinetics of the EOR electrooxidation reaction

**Fig 11:**
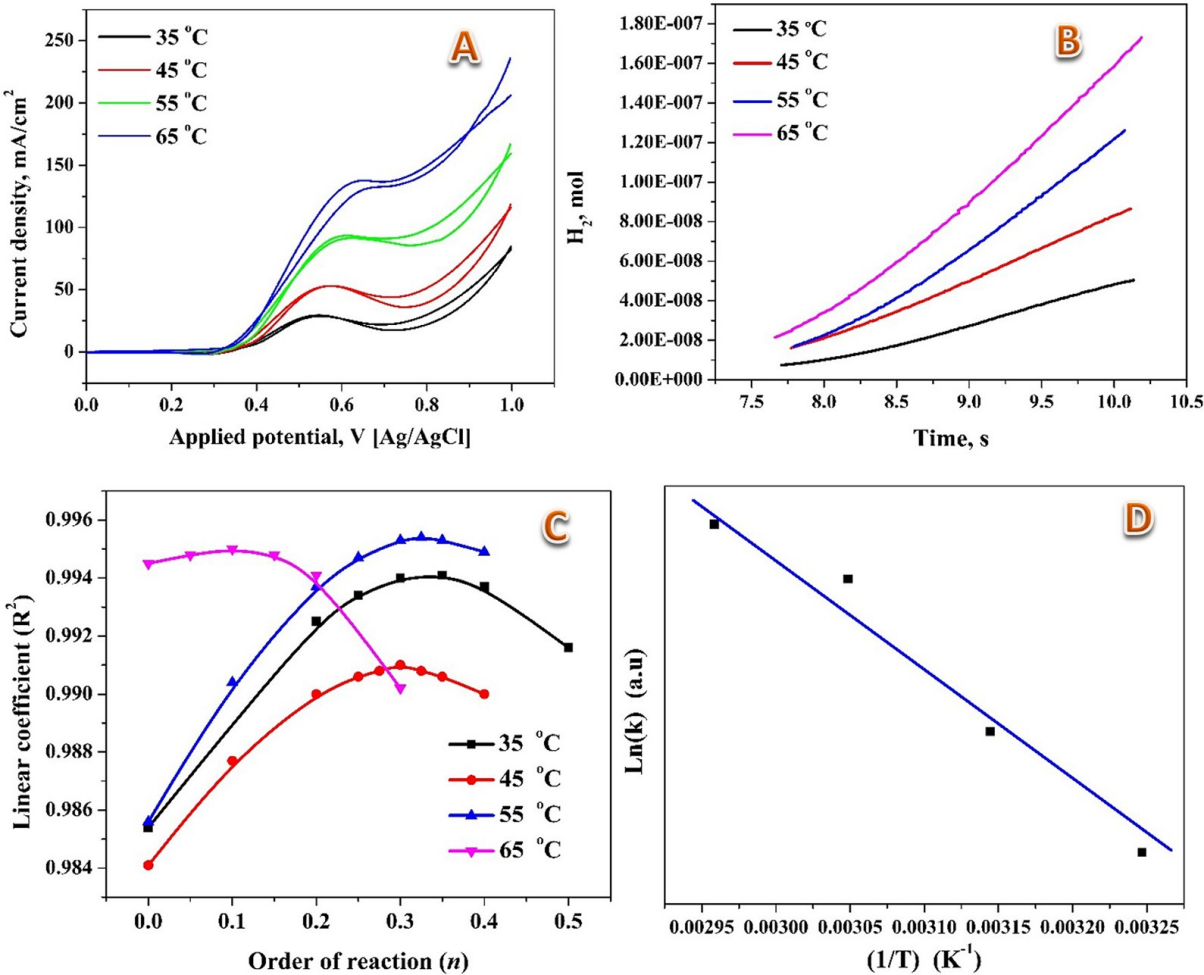
Effect of reaction temperature and the EOR process kinetics for 5% Mo/Ni NFs calcined at 850°C, 1 M ethanol concentration. A. Effect of reaction temperature, B. moles of hydrogen versus reaction time, C. *R*^2^ value versus order of reaction and D. Arrhenius plot, ln K versus 1/T.

In the realm of chemical reactions, the basic notion is that higher temperatures foster increased reaction rates, owing to the heightened kinetic energy of molecules in the system. This augmented kinetic energy translates to more frequent collisions per unit time, resulting in accelerated reactions. As illustrated in Fig 11A, outcomes from the electrooxidation of an ethanol solution (1.0 M) using the introduced catalyst at varying temperatures (35°C, 45°C, 55°C, and 65°C) reveal as expected trends. The figure demonstrates that the electrooxidation of ethanol rises with the temperature almost in a linear function. In other words, the current density at the oxidation peak increases linearly with the reaction temperature. The Eq 5 represents the fundamental ethanol electrochemical reaction involved:

$$CH_{3}CH_{2}OH+3H_{2}O\to CO_{2}+12\;H^{+}+12e^{‐}$$ (5)

Incomplete cracking of ethanol molecules can lead to the formation of various intermediates, as exemplified by reactions in Eqs 6 and 7, such as:

$$CH_{3}CH_{2}OH+3H_{2}O\to CH_{3}COOH+4H^{+}+4e^{‐}$$ (6)

$$CH_{3}CH_{2}OH\to CH_{3}CHO+2H^{+}+2e^{‐}$$ (7)

However, it is essential to emphasize that the total number of electrons generated in these intermediate reactions still equals the number of formed hydrogen ions. Hence, determining the produced hydrogen from the generated current is a valid and accepted approach. Faraday’s law of electrolysis, which relates the quantity of substance produced at an electrode during electrolysis to the quantity of electric charge passed through the electrode, supports this analysis.

Addressing the environmental implications associated with CO_2_ production during the electrooxidation of ethanol in an alkaline aqueous medium, a post-electrooxidation step will be proposed for capturing and utilizing the formed CO_2_ to ensure green hydrogen production. The CO_2_, being an acid gas, will readily react with NaOH, forming stable sodium carbonate compounds. Our forthcoming research endeavors will focus on developing efficient methods to separate and remove the generated sodium carbonate.

The kinetics of the chemical reaction start by studying the reaction rate (*r*); Fig 11B. The rate of reaction (*r*) can be approximated using the Equation:

$$r=\frac{dC_{A}}{dt}=kC_{A}^{n}$$ (8)

Here, [*C*_*A*_] denotes the concentration of produced hydrogen at time *t*, while *k* and *n* represent the rate constant and the reaction order of ethanol oxidation, respectively. It is worth mentioning that the produced hydrogen has been estimated from the detected current using ’ ’Faraday’s Equation. Rearranging Eq (8) yields:

$$\int_{C_{A0}}^{C_{A}}\frac{1}{C_{A}}dC_{A}=\int_{0}^{t}dt$$ (9)

Curiously, Eq (8) fails to hold at all temperatures, signifying that the reaction order does not remain constant across reaction temperatures. Consequently, Eq (9) estimates the reaction order at every temperature. Excepting *n =* 1, the integration can be algebraically determined as follow, Eq (10):

$$\frac{1}{1-n}C_{A}^{\left(1-n\right)}=kt+constant$$ (10)

The reaction order at every temperature could be estimated by estimating the R-squared (R^2^, the coefficient of determination) of the linear model regression at different values of *n*. As shown in Fig 11C, the reaction order slightly varies with the reaction temperature; Table 1 summarizes the reaction order, revealing the maximum *R*^*2*^ value.

**Table 1:** Order of reactions and linear model coefficient (*R*^2^) for EOR electrooxidation.

**Temperature, K**	308	318	328	338
**The order of reaction**	0.350	0.300	0.325	0.100
***R*** ^**2**^	0.941	0.991	0.9954	0.995

The data presented in Fig 11D led to the calculation of the activation energy of 33 kJ/mol using the Arrhenius equation. The Arrhenius equation is a fundamental concept in chemical kinetics that establishes a quantitative relationship between the rate constant of a reaction and the temperature at which the reaction occurs. This Equation is crucial for understanding how temperature influences reaction rates and provides insights into the energy barriers that reactions must overcome to proceed. The Arrhenius equation is expressed as follows, Eq (11):

k=Ae−EART (11)

Where: *k* represents the rate constant of the reaction. *A* is the pre-exponential or frequency factor related to the frequency of successful collisions between reactant molecules. *E*_*A*_ denotes the activation energy, the minimum energy required for reactant molecules to transform into products. *R* (8.314 J/mol∙K) represents the gas constant. *T* represents the absolute temperature (in Kelvin).

In the context of the obtained results, the calculated activation energy of 33 kJ/mol signifies the energy required for the ethanol oxidation reaction. A lower activation energy suggests the reaction can proceed more readily, even at lower temperatures. Conversely, a higher activation energy implies that the reaction requires a more considerable input of energy to overcome the energy barrier, making the reaction less favorable.

The calculated activation energy value of 33 kJ/mol holds important implications for the ethanol oxidation reaction studied. Activation energy measures the energy barrier that reactant molecules must overcome to initiate a chemical reaction [44]. This low activation energy suggests that the catalyst effectively lowers the energy barrier for the reaction, allowing it to occur more efficiently [45]. The obtained activation energy in our study represents a critical parameter that sheds light on the energetics of the ethanol electrooxidation process. Comparing this value with findings from previous studies further elucidates the impact of Mo_2_C in our nanofiber composite.

In the study by Barbasa et al. [46], where the electrooxidation of ethanol was investigated over a pristine nickel electrocatalyst, and an activation energy of 49 kJ/mol was reported. The higher activation energy in the absence of a co-catalyst suggests that introducing Mo_2_C in our composite catalyst is crucial in facilitating the ethanol electrooxidation process. This observation aligns with the general understanding that incorporating co-catalysts can reduce activation energy, as demonstrated in other catalytic systems. A relevant point of comparison arises from the work on Co-Ni-Mo/carbon fiber composite, where different compositions were studied. Activation energy values of 52.1, 38.1, and 39.3 kJ/mol were reported for co-catalyst contents of 60%, 40%, and 18%, respectively. These results further support our claim regarding the favorable role of the co-catalyst (Mo_2_C) in decreasing the activation energy. The trend of reducing activation energy with increased co-catalyst content is consistent with our findings, emphasizing the significance of Mo_2_C in promoting more facile ethanol electrooxidation kinetics.

### 3.4 Statistical manipulation and data analyses

**Fig 12:**
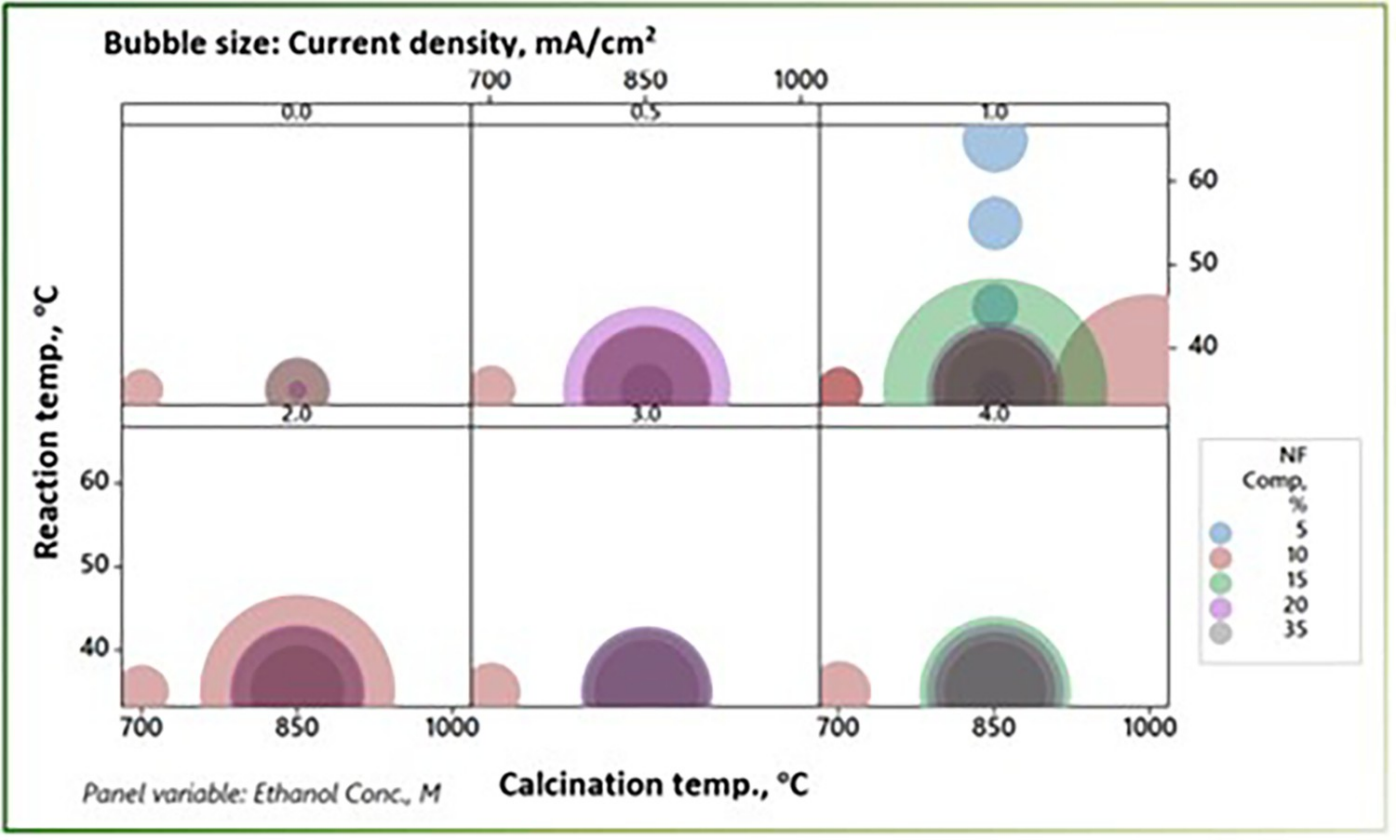
The bubbles diagram of the EOR process parameters influences where the bubbles’ diameters and colors represent the corresponding peak current density and the nanofibrous composite composition, respectively.

The different process parameters reflected their influences to different degrees. The bubbles diagram, Fig 12, summarizes, in a comparative theme, the influences of the EOR process parameters, with the balls’ diameters and colors representing the corresponding peak current Density and the nanofibrous composite composition, respectively. The diagram points out the low efficiency at the lowest calcination temperature at all other operating conditions. The same trend could be noted for ethanol concentration, with maxima occurring between 1 and 2 M. Moreover, the reaction temperature showed a semi-parabolic effect on the peak current density. The portrayed theme explores the effects of the different combinations of the process variables, hinting at the need and the anticipated room for optimization. The Spearman-Rho correlation was investigated for quantitatively mining the otherwise hidden effects among the process variables, Table 2. The correlation coefficients show that calcination temperature has a more significant effect on the PCD (coeff. 0.563 with a P-value ≈ 0), with the ethanol concentration having a moderate one (coeffs. 0.324 with P-value of 0.054). The reaction temperature was insignificant or had no correlation or effect on PCD, possibly due to the low number of experiments conducted at different levels of that variable.

**Table 2:** Spearman Rho correlations matrix for the EOR process.

	Ethanol Conc., M	NFC Comp., %	Reaction Temp., °C	Calcination Temp., °C
**NFC Comp., %**	0.089			
**P-value**	0.608			
**Reaction Temp.,** ^**o**^**C**	-0.089	-0.485		
**P-value**	0.605	0.003		
**Calcination Temp.,** ^**o**^**C**	-0.003	0.261	0.120	
**P-value**	0.986	0.124	0.485	
**Current density, mA/cm** ^**2**^	0.324	0.307	-0.251	0.563
**P-value**	0.054	0.068	0.139	0.000

Cell Contents: Spearman rho coeff. and P-Value

Table 3 summarizes the EOR parameters’ descriptive statistics and the peak’s current density as a response parameter. The data show that neither the variables nor the response follows the normal distribution of the current density. The variables were selected arbitrarily and biased by repeating one level of one variable many times in experimental runs, so it is intuitive not to follow the normal distribution with Skewness and Kurtosis values marking the direction and weight of deviation from normality.

**Table 3:** Summary of the descriptive statistics of the EOR variables and the current density as the response parameter.

Variable	Mean	StDev	Min	Q1	Median	Q3	Max.	Range	Skewness	Kurtosis	Anderson–Darling Normality Test	
											P-value	Decision
**Ethanol Conc., M**	1.653	1.308	0.00	0.625	1.00	3	4.00	4.00	0.65	-0.85	<0.005	Fail
**Reaction Temp.,** ^**0**^**C**	36.67	6.09	35.0	35.00	35.0	35	65.0	30.0	3.92	15.47	<0.005	Fail
**Comp., %**	15.97	9.70	5.0	10.00	10.0	20	35.0	30.0	1.10	0.04	<0.005	Fail
**Calcination temp.,** ^**o**^**C**	825.0	67.1	700.	850.0	850	850	1000.	300.	-0.79	1.18	<0.005	Fail
^*^ **PCD, mA/Cm** ^**2**^	52.75	48.73	1.00	11.84	43.94	72.0	210.7	209.7	1.43	2.30	<0.005	Fail

Number of experimental runs: 36; *: peak current density.

The general linear model (GLM) is chosen to fit the data since the response parameter, PCD, is continuous, including four covariates and their interaction and polynomial terms. The model was statistically driven, selecting the optimal λ for the Box-Cox transformation option, which showed an *R*^*2*^ of 78.33%, describing a moderate-to-poor fit of the model to the data. The reasons behind such low *R*^2^ values could be explained in terms of the unbalanced weight of the experiments at the different levels of the covariates. However, the mathematical expression is not the target output; it is the deep look at the inherent interactions otherwise hidden.

**Fig 13:**
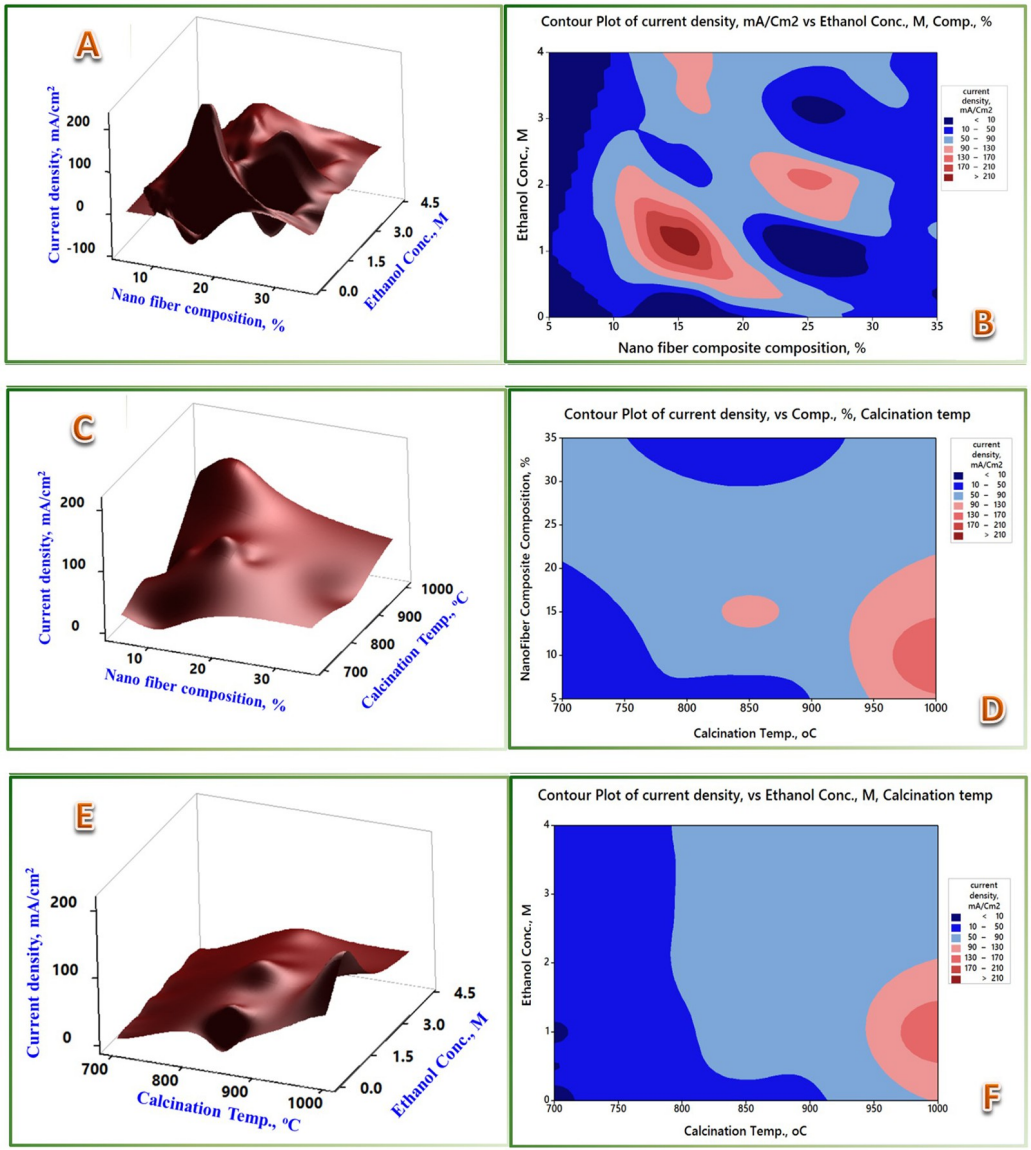
3D surface and contour plots of the EOR process covariates in pairs.

A portrayed visualization is provided through the 3D surface plots and contour plots in Fig 13. The theme in Fig 13A and 13B remarks on the strong interaction of ethanol concentration and nanofiber composition, pointing out that some troughs (i.e., degraded performance) may result in specific combinations of the two covariates. Those troughs, giving combinations, should be avoided and seek for those giving rise to the maxima. Somehow, a similar trend is noted for NFC and calcination temperature variables with an emphasis on the dominance of the calcination temperature; Fig 13C–13F demarcates the dominance of the calcination temperature parameter.

**Fig 14:**
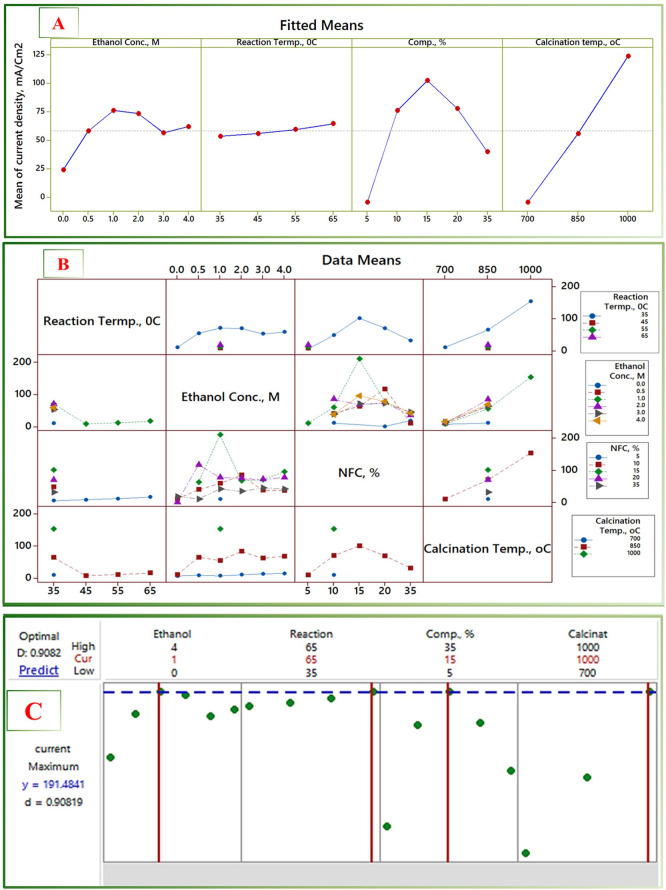
The Covariates’ main effects, interaction plots,3D surface, and the dynamic response optimizer plots. (A) Main effects plot, (B) interactions plot, (C) response optimizer.

The main effects plot, Fig 14A, shows the covariates’ relative effects and the response parameter’s sensitivity to the variations of the individual covariates. Calcination temperature explores steep linear direct effects on the response in contrast to the very mild one, almost horizontal, for reaction temperature covariate. The observed mild effect of the reaction temperature variable may be ascribed to the limited number of experiments carried out at different levels rather than the physical effect of the covariate on the process. The other two covariates, ethanol concentration and the nanofiber composite composition (NFC), showed maxima of clear inflection points. The slope of change in the case of NFC is much steeper, pointing out the higher sensitivity of the current density to the changes in this covariate and, hence, the benefits of its optimization. A thorough look at the paired interactions is shown in the interaction plot, Fig 14B. Those themes corroborate the inferences mentioned above. The reaction temperature explores no propagated mutual interactions as most experiments are carried out at the 35°C reaction temperature. The calcination temperature covariate demonstrates a clear dominance over the others, expressing each level on a parallel top-up line. The ethanol concentration and NFC covariates showed much stronger interactions, demarcating their room for the EOR process optimization. The 1 M ethanol concentration level and the 15% NFC demonstrate the maximum yield of peak current density.

The findings suggest a potential avenue for optimizing the process by manipulating the interdependent variables at specific levels to mitigate challenges that could arise from a particular factor level. For instance, if the electrocatalyst possesses an undesirably high Mo content (>10 wt.%), this limitation could be counterbalanced by operating at a moderate temperature while utilizing a higher ethanol concentration and conversely. Fig 14C graphically illustrates the optimal configuration using the response optimizer function. The identified optimal conditions involve a Mo content of 15 wt.%, an ethanol concentration of 1.75 M, and a reaction temperature of 59°C.

## 4. Conclusions

Good morphology nanofibers composed of nickel and molybdenum carbide NPs-incorporated CNFs can be synthesized from sintering of nanofibers containing nickel acetate tetrahydrate, molybdenum chloride, and polyvinyl alcohol at high temperatures under inert atmosphere. These nanofibers show excellent electrocatalytic activity toward ethanol electrooxidation in alkaline media. However, the molybdenum content strongly affects the electrocatalytic activity; 18.7 wt% is the optimum value. Moreover, increasing the calcination temperature distinctly improves the electrocatalytic activity. Compared to the Nanoparticulate morphology, the nanofibrous structure effectively improves the electrocatalytic activity toward ethanol oxidation. The statistical analysis of the experimental data employing a set of visualization-based tools and the general linear model (GLM) was found helpful, and it helped figure out the interrelationships among the interplaying factors and construct a dynamic optimization plot. The optimal conditions that generate the highest current density were identified as Mo content at 15 wt.%, methanol concentration of 1.55 M, and reaction temperature of 59°C. Generally, the study creates a new strategy to exploit molybdenum and the morphology of the nanofibers to synthesize effective non-precious electrocatalysts for alcohol electrooxidation for the sake of green hydrogen generation.
